# Tracking the Feeding Patterns of Tsetse Flies (*Glossina* Genus) by Analysis of Bloodmeals Using Mitochondrial Cytochromes Genes

**DOI:** 10.1371/journal.pone.0017284

**Published:** 2011-02-28

**Authors:** Catherine N. Muturi, Johnson O. Ouma, Imna I. Malele, Raphael M. Ngure, Jane J. Rutto, Klaus M. Mithöfer, John Enyaru, Daniel K. Masiga

**Affiliations:** 1 Molecular Biology and Biotechnology Department, International Centre of Insect Physiology and Ecology, Nairobi, Kenya; 2 Kenya Agricultural Research Institute, Trypanosomiasis Research Centre, Kikuyu, Kenya; 3 Tsetse and Trypanosomiasis Research Institute, Tanga, Tanzania; 4 Department of Biochemistry and Molecular Biology, Egerton University, Egerton, Kenya; 5 Department of Biochemistry, Makerere University, Kampala, Uganda; New Mexico State University, United States of America

## Abstract

Tsetse flies are notoriously difficult to observe in nature, particularly when populations densities are low. It is therefore difficult to observe them on their hosts in nature; hence their vertebrate species can very often only be determined indirectly by analysis of their gut contents. This knowledge is a critical component of the information on which control tactics can be developed. The objective of this study was to determine the sources of tsetse bloodmeals, hence investigate their feeding preferences. We used mitochondrial cytochrome c oxidase 1 (COI) and cytochrome b (cytb) gene sequences for identification of tsetse fly blood meals, in order to provide a foundation for rational decisions to guide control of trypanosomiasis, and their vectors. *Glossina swynnertoni* were sampled from Serengeti (Tanzania) and *G. pallidipes* from Kenya (Nguruman and Busia), and Uganda. Sequences were used to query public databases, and the percentage identities obtained used to identify hosts. An initial assay showed that the feeds were from single sources. Hosts identified from blood fed flies collected in Serengeti ecosystem, included buffaloes (25/40), giraffes (8/40), warthogs (3/40), elephants (3/40) and one spotted hyena. In Nguruman, where *G. pallidipes* flies were analyzed, the feeds were from elephants (6/13) and warthogs (5/13), while buffaloes and baboons accounted for one bloodmeal each. Only cattle blood was detected in flies caught in Busia and Uganda. Out of four flies tested in Mbita Point, Suba District in western Kenya, one had fed on cattle, the other three on the Nile monitor lizard. These results demonstrate that cattle will form an integral part of a control strategy for trypanosomiasis in Busia and Uganda, while different approaches are required for Serengeti and Nguruman ecosystems, where wildlife abound and are the major component of the tsetse fly food source.

## Introduction

Human African Trypanosomiasis (sleeping sickness) is a zoonotic disease with many endemic foci in sub-Saharan Africa, where it is spread by tsetse flies (*Glossina* genus). Transmission occurs largely among rural populations, where activities such as agriculture and fishing expose people to the bite of the tsetse fly and therefore the risk to infection with trypanosomes. Following large epidemics at the beginning of the last decade, which destroyed whole village communities in Central Africa [1], the number of reported new cases had reduced substantially by 2005, although unacceptable levels continued to be reported in Angola, the Democratic Republic of Congo and Sudan [2]. Livestock in endemic foci are constantly under threat of infection, especially where there is close proximity with wildlife. Animal trypanosomiasis (nagana) in Africa has largely excluded livestock from approximately 70% of the humid and semi-humid zones of sub-Sahara [3]. The most recent estimates of losses to Africa's Gross Domestic Product (GDP) are in the region of 4.5 billion US dollars per year, with about 3 million cattle deaths [4].

Information on what haematophagous insects feed on provides an important foundation on which to establish control strategies. Tsetse flies do not swarm and are therefore notoriously difficult to observe in nature. They larviposit 3rd instar larvae singly in the soil, and as adults, feed on a variety of vertebrate hosts in activity peaks and for short periods [5]. Hence since direct observation in the wild is virtually impossible, knowledge of their feeding habits and preferences can only be obtained indirectly. Additionally, indirect methods are necessary because of the terrain and fauna associated with tsetse fly infestation. To some extent, it is conceivable that tsetse flies feed on whatever suitable hosts are available in an opportunistic way, but when there is an abundance of choices, they select their host based on olfactory and visual cues [6]. Information on the source of blood meals of tsetse fly vectors is essential in understanding the relationship between hosts and vectors, and their respective roles in the trypanosomiasis transmission cycle [7]. Furthermore, blood meal analysis provides important information relating to the epidemiology of trypanosomiasis and natural feeding habits of different species of *Glossina*. It is known that each species of blood feeding arthropod feeds on a limited range of host species, with varying degrees of frequency on individual species, when given a choice [8]. This is the foundation on which development of olfactory attractants that are deployed in tsetse fly traps are based, being isolated from preferred hosts [9]. Attractants have been particularly effective for Savannah species of tsetse. Similar studies are being undertaken to develop attractants for riverine species. For example, Omolo and colleagues [10] report significant increases in catches of subspecies of *Glossina fuscipes*, in the presence of monitor lizards (for *G. f. fuscipes*) and pigs (*G. f. quanzensis*). Serological techniques for identifying tsetse fly bloodmeals, including precipitin and haemagglutination test [11], complement fixation test [12] and enzyme-linked immunosorbent assays (ELISA) [13], [14] have been applied with reasonable success. However, standardized commercially available reagents do not exist, and there is considerable difficulty distinguishing closely related species [13]. Short conserved regions of mitochondrial cytochrome b (cytb) applied as a heteroduplex PCR assay [15], and combined with RFLP [16] provided better resolution. In this study, we provide an extensive evaluation of a new approach which uses a 648 bp fragment at the 5′ end of cytochrome c oxidase 1 (COI) [17] as a DNA barcode for species identification for which there is a rapidly growing database to facilitate identification (www.barcodinglife.org; [18]. We complement this with the analysis of cytb.

## Methods

### Study sites and sample collection

**Figure 1:**
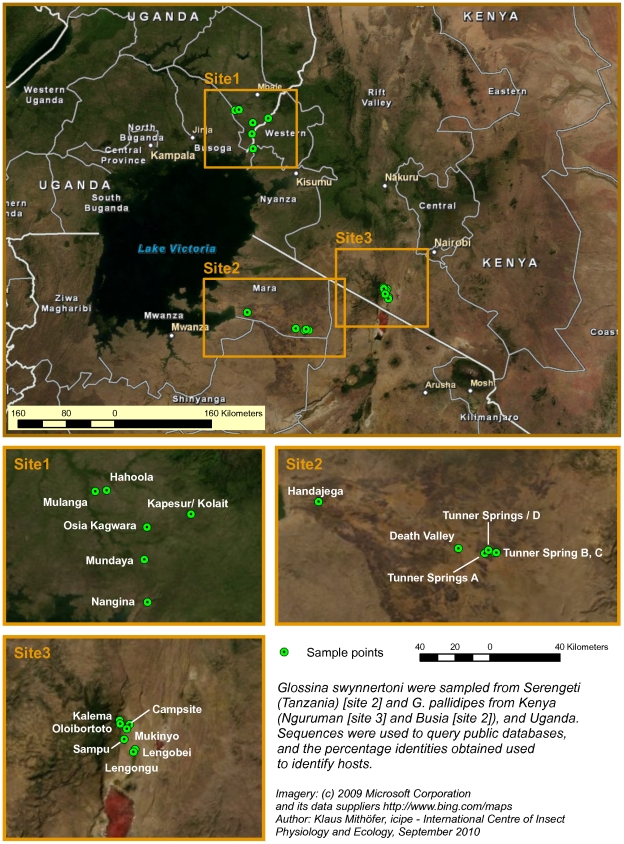
A map showing georeferenced sites where tsetse flies were trapped in Kenya, Tanzania and Uganda. A Google Earth map is also available (http://www.icipe.org/images/stories/downloads/muturi_et_al_figure1.zip.zip).

Tsetse flies were trapped in 2008 and 2009, at the sites shown in Figure 1. In all locations, tsetse flies were caught using biconical traps [19] baited with acetone and cow urine [20]. Trapping was done in the Serengeti National Park (northwestern Tanzania), Nguruman (southwestern Kenya), Busia (western Kenya/eastern Uganda) and northeastern Uganda. In the Serengeti Ecosystem, flies were trapped at Death Valley (DV), Tunner Spring (TS) and Handajega (HA). Features that characterize Death Valley include a mixture of open woodlands, dense woodland, savannah and grassland. The Tunner Spring area consists of open grassland and few thickets in between whereas Handajega is an open savannah intermingled with open woodlands and very close to human settlement. The trapping areas in Nguruman were near the escarpment, where two tsetse species, *G. pallidipes* and *G. longipennis* have been recorded, with the former being the predominant species [21]. The vegetation consists of patches of lowland woodland surrounded by open savannah, through which tsetse disperse during wet seasons [22], and there is abundant game [23]. The Busia area in western Kenya, eastern and northern Uganda are agricultural areas with mixed crop and livestock farming. There are no significant populations of large wild mammals. In all localities, blood-fed flies were identified using morphological keys [24] and either preserved whole in 95% ethanol, or midguts squashed onto Whatman No.1® filter paper that were then kept desiccated with silica gel. Both were stored at 4°C within 24 h of collection until processed.

### DNA extraction

Midguts were dissected out of ethanol preserved flies and DNA extracted using QIAgen DNeasy extraction kits following the manufacturers instructions (QIAgen inc., Valencia, CA, USA). For Whatman filter papers, discs of about 2 mM diameter were cut out of the blood spots on the filter papers and placed into microcentrifuge tubes containing extraction buffer (10% SDS, 0.15 M NaCl, 50μg/ml proteinase K and 0.1 M EDTA), and incubated at 56°C for 2 hours. The mixture was then extracted with phenol-chloroform-isoamyl alcohol using standard methods [25].

### Amplification and sequencing

A 648-bp target region of the 5′ end of the mitochondrial cytochrome oxidase *c* subunit 1 (COI) gene [17] was amplified using the primers with a broad target group including mammals, reptiles and fishes VF1d_t1 (5′-TGTAAAACGACGGCCAGTTCTCAACCAACCACAARGAYATYGG-3′) and VR1d_t1 (5′-CAGGAAACAGCTATGACTAGACTTCTGGGTGGCCRAARAAYCA-3′) [26]. Initial denaturation was done for 2 min at 94°C, followed by 40 cycles of denaturation at 94°C for 30 s, annealing at 45°C for 45 sec, and primer extension at 72°C for 1 min followed by a final extension at 72°C for 10 min. PCR was carried out in a total volume of 25 μl containing 10pmol of each primer, 10 mM Tris-Cl, pH 8.3 and 50 mM KCl, 1.5 mM MgCl_2_, 2.5 mM dNTPs, 2μl of the DNA template and 1unit *Taq* DNA polymerase (Genscript Corp, Piscataway, NJ). The 359 bp fragment from mitochondrial cytochrome b (cytb) fragment was recovered using primers Cb1 (5′-CCATCCAACATCTCAGCATGATGAAA-3′) and Cb2 (5′-GCCCCTCAGAATGATATTTGTCCTCA-3′), designed to cover the same amplicon region described for mammals [27]. Amplifications were carried out in 25μl volumes, with the same reagent profiles as with COI. Cycling was initiated by denaturation at 94°C for 5 minutes, followed by 35 cycles of denaturation at 94°C for 30 s, annealing at 55°C for 45 sec, and primer extension at 72°C for 30 s, with a final extension at 72°C for 10 min. Amplicons were column purified using the Quickclean 5 M PCR purification kit following manufacturer's instructions (Genscript Corp, Piscataway, NJ). Sequencing was outsourced, and done bidirectionally using the amplification primers. COI and cytb sequences were submitted to GenBank using Bankit, the web-based data submission tool (www.ncbi.nlm.nih.gov/BankIt) [28].

### Sequence analysis

Consensus sequences were generated by contiguation functions of BioEdit, Version 7.0.9 [29] or Geneious Version 5.0.2 [30] and manually edited, where necessary, by reference to the chromatograms. Consensus sequences of approximately 648 bp for COI, and 320 bp for cytb were generated. Cytb species assignments were based on querying GenBank non-redundant database using BlastN [31]. COI species assignments were done using the identification engine at the Barcode of Life Data systems (BOLD: www.barcodinglife.org) [18], with species-level identifications based on sequence identity of at least 99% to designate bloodmeal sources. A similar approach was used to identify insect fragments in fecal samples of the eastern red bat (*Lasiurus borealis*) [32], but these authors used phylogenetic analysis in addition.

## Results

### Species identifications

An initial assay showed that most feeds were from single sources, and we did not find any chromatogram profiles with ambiguous nucleotide bases that suggested multiple bloodmeal sources. Out of thirteen engorged tsetse flies (*G. pallidipes*) caught in the Nguruman area six (6) had fed on *Loxodonta africana* (African savanna elephant), five (5) on *Phacochoerus africanus* (warthogs), while one fly each had fed on *Syncerus caffer* (African buffalo) and *Papio hamadryas* (baboon). All twelve (12) flies (*G. pallidipes*) sampled in Busia (eastern Uganda/western Kenya) had fed on *Bos taurus* (cattle). Hosts identified from forty (40) blood-fed *G. swynnertoni* in the Serengeti ecosystem had fed on *Syncerus caffer* (African buffalo), which accounted for twenty-five (25), while eight (8) had fed on *Giraffa camelopardalis* (giraffe), three (3) each on *Phacochoerus africanus* (warthog) and *Loxodonta africana* (African savanna elephant), while one had fed on C*rocuta crocuta* (spotted hyena). Identification of bloodmeals from *G. swynnertoni* was based entirely on analysis of cytb, since the COI primers used only gave amplicons of *G. swynnertoni*. Table 1 gives details of the identifications and GenBank accession numbers for COI and cytb.

**Table 1:** Summary of data for bloodmeal analysis showing species fed on by tsetse flies caught in the different study sites.

Species Identified	Nguruman (n = 13)	Mbita Point (n = 4)	Busia & N. Uganda^1^ (n = 12)	Serengeti (n = 40)	GenBank accession numbers (COI)	GenBank accession numbers (cytb)
African Savannah Elephant (*Loxodonta africana*)	6	0	0	3	HQ219070, HQ219071, HQ219072	HQ634690, HQ634691, HQ680406
Warthog (*Phacochoerus africanus*)	5	0	0	3	HQ219073, HQ219074	HQ634692, HQ634693, HQ680407
Nile Monitor lizard (*Veranus niloticus*)	0	3	0	0	HQ219067, HQ219068, HQ219069	ND
Cattle (*Bos taurus*)	0	1	12	0	HQ219063, HQ219064, HQ219065, HQ219066	HQ634689, HQ680403, HQ680404, HQ680405
African Buffalo (*Syncerus caffer*)	1	0	0	25	ND	HQ634694, HQ680408, HQ680409, HQ680410, HQ680411, HQ680412HQ680413, HQ680414, HQ680415, HQ680416, HQ680417, HQ680418HQ680419, HQ680420, HQ680421, HQ680422, HQ680423, HQ680424HQ680425, HQ680426, HQ680427, HQ680428, HQ680429, HQ680430
Giraffe (*Giraffa camelopardalis*)	0	0	0	8	ND	HQ634695
Spotted Hyena (C*rocuta crocuta*)	0	0	0	1	ND	HQ680402
Baboon (*Papio hamadryas*)	1	0	0	0	ND	HQ680401

The total number of flies analyzed per site is given in parenthesis. These data combine both cytb and COI, and GenBank accession numbers for representative sequences given, and where not determined indicated as ND.

1. These data include 2 samples from *G. pallidipes* in northern Uganda.

## Discussion

In the present study we used mitochondrial cytochromes as molecular targets for identifying sources of tsetse fly bloodmeals. The approach we took to use generic primers for PCR is arguably the most viable method for determining the feeding hosts of tsetse flies. Although valuable data have been obtained using ELISA methods (for example, [14]), this approach requires prior development of antisera against all possible sources of bloodmeals in the locality where tsetse flies are caught. Except for the Serengeti National Park in Tanzania, the numbers of tsetse flies trapped were quite small, consistent with previous observations that tsetse sampling tools are biased towards hungry flies [33], and field samples usually contain little of their last blood meals in their midgut still present [34].

Generic primers for COI [26] designed to amplify the 5′ end of the gene in templates prepared from mammals, fishes and reptiles were used, while those described by Kocher et al [27] were applied for cytb. For *G. swynnertoni* caught in Serengeti, each PCR product obtained for COI was tsetse, not vertebrate origin. Considering that the same protocol was applied to all samples, we presume that the primers anchored better to tsetse than vertebrate DNA. This was not investigated further, but more specific primers better suited to discriminate vertebrate from tsetse DNA will need to be designed. For Serengeti therefore, only cytb data were used. We did not find any evidence of mixed feeds (multiple host species), which is detectable by analysis of DNA sequences [35]. Perhaps tsetse frequently feed to completion on one host, or it may be that in a homogenous herd flies hoping from one host to another would be indistinguishable from those feeding on a single animal. It is also possible that in a mixture where one bloodmeal source is predominant, the PCR process could mask the minor one as the more abundant one may be preferentially amplified.

Nguruman and Serengeti are areas where wildlife is protected by national law, but which also afford interaction with livestock farmers. However, drought often forces farmers to move their livestock to other areas, sometimes out of the tsetse fly belt. Hence, in Nguruman and Serengeti, wildlife is vital for the survival of the tsetse fly population, hence maintaining the trypanosome pathogens that cause trypanosomiasis. Previous studies in both Nguruman and Serengeti, undertaken using ELISA found elephants and warthogs among the most common blood meal sources [36], [37]. In these areas, an effective approach to control the disease must target reducing the numbers of tsetse, for example through spraying with insecticides using suitable aircraft, the strategy used to eliminate *G. morsitans centralis* from the Okavango Delta in Botswana [38], or deploying odor-baited traps and targets, the latter impregnated with insecticides. It is known that in and around the Serengeti National park, parasites that cause Human African Trypanosomiasis (HAT, or sleeping sickness) may persist in both domestic livestock and wildlife reservoirs [39]. In Busia, domestic livestock areas important hosts for tsetse flies and harbor human infective forms of trypanosomes [40]. Several studies have shown the efficacy of treating cattle with insecticides as mobile targets can lead to the reduction in tsetse populations (http://www.researchintouse.com/nrk/RIUinfo/PF/LPP14.htm), helping to break the vector link between parasite reservoirs and humans.

The Serengeti and Nguruman provided the most diversity of bloodmeal sources, not surprisingly considering that both areas have abundant wildlife. Blood from large mammals (buffalo, elephant and giraffe) were a significant component of the tsetse fly diet. There were some surprises in the data obtained. No tsetse flies were found to have fed on antelopes, in Serengeti and Nguruman, where species of these abound, despite knowledge that antelopes can account for more than 30% of bloodmeals [15]. Also, no cattle were identified in the Nguruman bloodmeals. This may be attributed to the prolonged drought during the sampling period, and the semi-nomadic Maasai herdsmen had moved the livestock to areas with pasture.

Knowing what the preferred hosts of bloodmeals can provide a more targeted approach to finding olfactory cues to improve efficiency of tsetse traps, which remains an active field of study [10], [41]. While matching DNA sequences to databases can be excellent tools for identification of species, they depend on the quality of representation as DNA sequences in the database [42]. This study illustrated the utility of COI and cytb gene sequences for the identification of the vertebrate hosts of the tsetse fly species. A larger scale study is required to include more hosts to enable development of methods such as PCR-RFLP, which can circumvent the need for sequencing.

Are tsetse flies generalists or specialists? Will they feed on anything available, or are they choosy when presented with options? On the basis of this study, the size of the host matters, presumably because visual cues are a critical complement to olfaction, as does what fauna are available.
